# Ventilation of Rooms for Sick*Read before Friend’s Literary and Library Association of New York, February 18, 1886.

**Published:** 1886-03

**Authors:** C. H. Bushong

**Affiliations:** 337 West 50th St.


### VENTILATION OF ROOMS FOR THE SICK.*

By C. H. Bushong, M. D.

A few remarks on general ventilation are appropriate as an introduction to the subject under consideration. The importance of this subject has been recognized only a short time. Since it has been agitated and has come to be generally known, a new profession has been ushered into being—that of sanitary engineer. These, while they differ widely in many things, all agree on two points, namely:—First: The necessity of a means for getting in plenty of new, pure air, to make a building a healthy habitation for man. Second: That infinitely few modern dwellings possess adequate means for furnishing this supply.

Lack of pure air in dwellings is a direct result of our advancing civilization, a result, we should say, that now bids fair soon to be a thing of the past. Until recently the main object in planning houses has been to make them as nearly air-tight as possible. They protect their occupants better from the vicissitudes of climate than those of a century or more ago, but they also give them almost no ventilation. They give more comfort with less healthful surroundings; luxury at the expense of purity of lung food. A dwelling made tight enough to keep cold air out will also keep foul air in, unless some contrivance to allow it to escape is also used. The houses of our ancestors, being less tight, allowed a free passage of air out and in, and their occupants were exempt from many modern diseases that we now know to be due to confinement in the foul air of close rooms. In this respect, our forefathers were better off than we are.

If we read the signs of the times aright, the era of unventilated houses will soon be ended. The investigations of scientists have borne fruit, and as the people obtain a more thorough knowledge of the results of their labors, they will become in an equal degree averse to living in air-tight houses. The discovery of spores, bacteriae, and poisonous gases in contaminated air has begun a revolution in house building. The dissemination of this knowledge has made, and will make, peopie demand that their homes shall be free from these deleterious substances. This demand is even now so general that builders dare not make dwellings without some device to give at least the appearance of ventilation, It is true that many of these contrivances are put in merely to allay the apprehensions of the prospective occupants, and are totally inadequate for the purpose ; yet, the fact that the people demand something is a healthy sign. It gives foundation for a hope that, in the near future, we shall have all homes furnished with easy and ample means for giving the occupants that abundance of pure, clean air, that is so needful for a condition of perfect health.

Many of our modern diseases are directly due to the re breathing of impure air. Some dwellings are pest houses for the accumulation and retention of atmospheric impurities. In order to have health we must drive out the tainted air, with its noxious gases, products of bodily waste, and disease germs, and supply an abundance of fresh air to take its place.

The oxygen, so necessary to life, comes from the atmosphere, a vast ocean of air that surrounds the earth. This is about one-fifth oxygen, and nearly four-fifths is nitrogen, an inert diluent. Every time air is taken into the lungs a portion of the oxygen passes through the thin walls of the air cells (vesicles) directly into the blood. In return, the blood gives up carbonic acid gas, (a poison,) and many impurities in the form of cast off waste products, for which the system has no further use. The expired air is thus injured in two ways. The nourishing principle is deficient, and the poisonous elements are increased. It can injure both in the way of not affording enough oxygen, and also by returning into the system a lot of hurtful things of which it has made an effort to rid itself by casting them off.

When the individual is sick all these conditions are much aggravated. The need of an abundance of pure air is much greater. Indeed many wasting diseases are now successfully treated by the inhalation of oxygen in larger amounts than the best of atmospheric air will give. The system needs every external aid in the form of nourishment that it can have to enable it to resist the inroads of the disease. In addition to this need of healthy food for the lungs, we must remember that, in most diseased conditions, the patients are much more susceptible to the influences of injurious surroundings.

Also, that the products of waste cast off into the air are greater in amount from sick than from well persons. To this must be added the increased amount constantly being cast off by the skin and taken up by the surrounding air, and also, in many cases, the disease germs that are constantly being thrown off. All these things combine to make the air of an unventilated sick room a very unclean and dangerous thing to take into one's lungs.

The object of ventilating sick rooms is to furnish means both to remove the bad air, and to let in an abundance of good air; by this means the impurities are kept in so dilute a form as to do a comparatively small amount of injury either to the patient or the nurse.

I have devoted a seemingly large amount of space to this part of my subject for a purpose. Few, except physicians and professional nurses, seem to realize how needful pure air is to the sick and the evils that are sure to follow a neglect in supplying it. The necessity of a well ventilated sick room is one of those truths that needs to be dwelt upon and expounded over and over again and then will be frequently neglected.

The idea of making the air in a room pure and nourishing without changing it has a strong hold on many. Some, even, who should know better, claim that this can be done. There are various ways advocoted by these theorists. Some are in favor of burning various substances in the room, others think it best to expose some volatile substance in the room and allow it to evaporate, and so on. They all claim that their favorate plan will kill the disease germs, etc., that are in the air. But it is a fallacy. If used in sufficiently dilute form to be without injury to the human beings in the room, they will also fail to destroy the other creatures. Then if they did what would it avail us? If we could destroy all the poisonous substances the air might be pure in that sense, yet be devoid of the needed amount of oxygen. There is no means of obtaining this except from without. It may often be wise to use some substance to conceal bad odors when their presence is unavoidable, but we must always remember that the object of this is only to conceal this evidence of the unwholesome presence, and that it does *not* destroy anything. It will not remove the poisonous elements, and it *cannot replace the oxygen* that is constantly being consumed by the patient and the nurse.

When a member of the family is taken sick and is to be cared for in the house, the first thing to see to is the room in which he is to live while the disease lasts. In order that he may have the best chances of a favorable termination of his sickness a number of things must be considered in deciding this question. The aim should be to choose the room which offers the greatest number of the requirements of a model sick room.

This room should be a large and cheerful one on a sunny side of the house. It should be in a quiet part of the house, away from the street and also from that part occupied by the rest of the family, so as to exclude all noise, and smells of cooking, etc. A second or third story is best. It should have a high ceiling, and at least two large windows. These should be on opposite sides of the room, or, at least, on two sides. They should have window-boards, or some contrivance to cause the incoming air to be reflected upward, and thus have an opportunity to get warmed before it reaches the patient. It must be remembered that warm air is not, of necessity, impure any more than cold air is all pure. An abundant supply of warm pure air is the object of ventilation.

If windows of sufficient size, or in proper places, can not be had, the same result can often be obtained by using an adjoining room with windows and a communicating door. If the weather is warm this door may be kept open. In cold weather it can be closed while the windows are opened to fill the room with pure, cold air, then the windows should be closed, and after the roomfull of fresh air has been warmed the door can be opened to let it in. This plan can be repeated as often as is deemed necessary.

It is sometimes an advantage to cover the patient thoroughly with heavy quilts and then throw open all the windows and doors and allow the air to pass freely through the room. This can be safely done, even in cold weather, provided care is taken not to uncover the patient until the room has been warmed sufficiently.

In this connection I will state that people with febrile diseases do not take cold easily while they have the fever, hence it is not necessary to bundle them up so that they are uncomfortable at such times. But it must be remembered that they do very readily take cold while convalescing from such diseases. Care is especially needed after a case of pneumonia, as relapses are due to a too early exposure in many cases where they occur.

Some nurses seem to think that if the air in a room has been changed once or twice in a day that it has been sufficiently renewed. Prof. Edward Curtis, of New York, says: "There should be three thousand cubic feet of outside air let in *each hour* for each pair of lungs." That means six, or more, thousand cubic feet of air for one patient and the nurse, and it must be renewed each hour. I find that a room eighteen feet long, eighteen feet wide, and eighteen feet high, contains a little less than six thousand cubic feet (5,832). As most rooms are much smaller than this the deficiency must be made up by renewing the air more frequently.

The best way to get foul air out of a room is by the way of the chimney. The contaminated air is heavy, therefore it is next the floor. The opening for its escape must, consequently, be low in the room. To cause this heavy air to go out, with its load of impurities, it must be heated. The best arrangement for this is a grate fire. It gives the needed ventilation, it also warms the room by radiation and it is cheerful.

If there is no grate there should be an opening low in the wall, either communicating with a chimney or with an air flue that is near enough to the chimney to be warmed by it. It is not sufficient to have an opening and a flue, it must be warmed so as to cause an upward current of air to pass out, and thus *draw* the impure air out of the room.

If sick rooms were more generally provided with proper ventilating facilities, fewer fatal terminations of the sickness would be the result. Many deaths that are now attributed to providence or the physician's want of skill, are really due to improper surroundings.

The sickness of those who have acted as nurses is often due to a long confinement in a room full of disease germs and other injurious substances given off by the patient. This danger is very materially diminished by attention to the proper ventilation of the room.

337 *West* 50*th St.*

* Read before Friend's Literary and Library Association of New York, February 18, 1886.

